# *Lactobacillus gasseri* SBT2055 reduces postprandial and fasting serum non-esterified fatty acid levels in Japanese hypertriacylglycerolemic subjects

**DOI:** 10.1186/1476-511X-13-36

**Published:** 2014-02-19

**Authors:** Akihiro Ogawa, Yukio Kadooka, Ken Kato, Bungo Shirouchi, Masao Sato

**Affiliations:** 1 Laboratory of Nutrition Chemistry, Department of Bioscience and Biotechnology, Faculty of Agriculture, Graduate School of Kyushu University, 6-10-1 Hakozaki, Higashi-ku, Fukuoka 812-8581, Japan; 2 Milk Science Research Institute, Megmilk Snow Brand Co. Ltd., 1-1-2 Minamidai, Kawagoe, Saitama 350-1165, Japan

**Keywords:** Probiotics, *Lactobacillus gasseri* SBT2055, Oral fat-loading test, Postprandial lipid response, Fermented milk products, Non-esterified fatty acid, Triacylglycerol

## Abstract

**Background:**

*Lactobacillus gasseri* SBT2055 (LG2055) inhibits dietary fat absorption in rats and exerts preventive effects on abdominal adiposity in rats and humans. The present study aimed to evaluate the effects of LG2055 on postprandial serum lipid responses in Japanese subjects with hypertriacylglycerolemia after the intake of oral fat-loading test (OFLT) meals.

**Methods:**

We conducted a single-blind, placebo-controlled, within-subject, repeated-measure intervention trial. Twenty subjects initially ingested the fermented milk (FM) without LG2055 for 4 weeks (control FM period), followed by a 4-week washout period, and then consumed FM containing LG2055 for 4 weeks (active FM period). The subjects were asked to consume FM at 200 g/day. At the end of each 4-week period, an 8-h OFLT was conducted. Blood samples were collected at fasting and every hour for 8 h after OFLT meal intake. Thereafter, postprandial serum non-esterified fatty acid (NEFA) and triacylglycerol (TAG) levels and fasting blood parameters were measured.

**Results:**

The OFLT showed that the postprandial serum NEFA levels from 120 to 480 min and the postprandial serum TAG level at 120 min in the active FM period were significantly (*P* < 0.05) lower than those in the control FM period. The fasting serum NEFA level in the active FM period significantly (*P* < 0.001) decreased at week 4 from the initial period compared with the control FM period.

**Conclusions:**

The consumption of probiotic LG2055 reduced postprandial and fasting serum NEFA levels, suggesting its possible contribution to the reduction of the risk for obesity and type 2 diabetes mellitus.

**Trial registration:**

UMIN000011605

## Background

*Lactobacillus gasseri* SBT2055 (LG2055), a probiotic lactic acid bacterium that originates from the human intestine [1,2] and has the ability to improve the intestinal environment [3], was demonstrated to diminish lymphatic triacylglycerol (TAG) transport and increase fecal fatty acids excretion, thereby inhibiting intestinal fat absorption [4], and to have anti-obesity effects in rats [4-6], mice [7], and humans [8,9]. In our previous clinical study [8,9], Japanese adults with obese tendencies consumed fermented milk containing LG2055 at 200 g/day for 12 weeks and consequently exhibited significantly reduced visceral fat areas, body weight, body mass index (BMI), and waist and hip circumferences. However, the effects of LG2055 on postprandial lipid metabolism remain to be investigated.

Several possible factors affect postprandial lipid metabolism. Energy intake reduction by suppression of dietary fat absorption is a strategy for the regulation of postprandial lipid metabolism. Orlistat, which is a strong lipase inhibitor [10] approved by the US Food and Drug Administration as a medication for long-term obesity treatment [11], improved postprandial lipid metabolism in overweight type 2 diabetic patients via the inhibition of fat absorption [12]. Functional food components were also shown to have a suppressive effect on fat absorption and an anti-obesity effect. For example, mannooligosaccharide (MOS) supplementation reduced fat absorption [13,14], leading to body weight and visceral fat loss after 12 weeks in humans [15,16]. The inhibition of dietary fat absorption by MOS was more moderate than that by orlistat; orlistat administration reduced fat absorption by approximately 30% [17], whereas MOS intake significantly lowered fat utilization by approximately 2% [14]. However, to our knowledge, no intervention study has been conducted to examine postprandial lipid metabolism using probiotics.

In this study, we examined the effects of the probiotic LG2055 on postprandial serum lipid responses in Japanese subjects with hypertriacylglycerolemia, referring to an oral fat-loading test (OFLT) study of Lopez et al. [18].

## Methods

### Subjects

Twenty adults, whose background characteristics are summarized in Table 1, were enrolled in this study. We recruited adults who had hypertriacylglycerolemia with fasting TAG levels higher than 200 mg/dl [19] and normal blood fasting glucose levels lower than 110 mg/dl. Those who had severe internal organ disorders, including coronary heart disease, renal impairment, hypothyroidism, or liver dysfunction, and hypersensitivity to dairy products were excluded. None of the subjects used tobacco, consumed special health-promoting foods, or took medication known to alter gastric emptying, lipoprotein metabolism, insulin secretion, or insulin activity.

**Table 1:** Baseline characteristics of the study subjects

**Parameters**		
Number of subjects	20	
Male	15	
Female	5	
Age (years)	51.1	(6.6)
Height (cm)	169.6	(10.5)
Body weight (kg)		
Male	73.7	(10.0)
Female	61.3	(6.3)
BMI (kg/m^2^)		
Male	24.2	(2.5)
Female	25.5	(1.4)
Systolic blood pressure (mm Hg)	111.0	(11)
Diastolic blood pressure (mm Hg)	73.0	(9)
Pulse rate (beats/min)	69.0	(8)
TAG (mg/dl)	277.0	(57)
Total cholesterol (mg/dl)	215.0	(41)
NEFA (mEq/l)	0.58	(0.22)
Glucose (mg/dl)	90.0	(7)
Insulin (μU/ml)	7.3	(2.1)

*Abbreviations*: *BMI* body mass index, *TAG* triacylglycerol, *NEFA* non-esterified fatty acid.

The data are expressed as mean (SD) values.

### Study design

This study was performed as a single-blind, placebo-controlled, within-subject, repeated-measure intervention trial. The crossover design was not adopted because it was possible that LG2055 survived in the intestinal tract after the washout period [3]. In a previous study, LG2055 was detected in a fecal sample even 90 days after LG2055 administration was discontinued [3]. Therefore, we set the placebo treatment period before the LG2055 treatment period. This study was conducted according to the guidelines established in the Declaration of Helsinki and the Ethical Guidelines for Epidemiological Research (Ministry of Health, Labour and Welfare of Japan), and all procedures involving human subjects/patients were approved by the institutional review board of Fukuda Clinic (Yodogawa-ku, Osaka, Japan) before initiation of the study. All the subjects provided written informed consent before participation in the study. This study was conducted from November 2011 to February 2012 by a contract research organization, Soiken Inc. (Toyonaka, Osaka, Japan). The clinical trial was registered in the University Hospital Medical Information Network Clinical Trials (No. UMIN000011605).

### Preparation of the test fermented milk

*Lactobacillus gasseri* SBT2055 (LG2055) was provided in fermented milk (FM). Two types of FM were prepared as previously reported [8], that is, active FM containing LG2055 and control FM lacking LG2055. The active FM was prepared with lactic acid bacteria starter cultures (*Streptococcus thermophilus* and *Lactobacillus delbrueckii* spp. *bulgaricus*) that are commonly used for conventional yogurt production and viable cells of LG2055. A FM mixture consisting of approximately 11% skim milk powder and a small amount of flavoring, agar, and sucralose as artificial sweetener with a zero-energy value was inoculated with the yogurt starter cultures and LG2055 cells and then cultured at 40°C for 3.5−4 h. The viable cell count of LG2055 was approximately 5 × 10^10^ cfu/100 g of FM on the initial day. The control FM was prepared in the same manner except that LG2055 cells were not added. These FM preparations were equivalent in energy (146.4 kJ), protein (3.7 g), fat (0.1 g), carbohydrate (4.9 g), and sodium content (41 mg) per 100 g and were indistinguishable in taste. The test FM preparations were kept in cold storage and delivered weekly.

### Study schedule and protocol

The subjects initially consumed the control FM for 4 weeks, followed by a 4-week washout period, and then the subjects consumed the active FM for 4 weeks. Initiation of the consumption of either the control or active FM was designated as week 0 (W0). While maintaining their habitual mode of living including diet and exercise, the subjects consumed FM at 200 g/day as two portions of 100 g each after breakfast and dinner.

Measurements of body weight, waist circumference, blood pressure, pulse rate, and fasting blood parameters and an interview with a physician were performed at the beginning and end of each 4-week intake period (W0 and W4, respectively). At the end of each 4-week intake period, an 8-h OFLT was performed. Each subject made a daily record of the amount of test FM intake, alcohol consumed, and medicine taken. A detailed dietary record was also made by the subjects for three consecutive days before each time point (W0 and W4). Information regarding subjective symptoms such as headache, nausea, and abdominal pain was collected through an interview with a physician at each time point (W0 and W4).

### Oral fat-loading test

The subjects stayed at the same hotel on the day prior to the OFLT and consumed a dinner with an energy content of approximately 3 765.6 kJ between 19:30 and 20:00 hours, after which the subjects were not allowed to eat and drink anything except water until the beginning of the OFLT. The total energy provided by the OFLT meals was 2 468.6 kJ, with a macronutrient profile of 72% fat, 24% carbohydrate, and 4% protein, which was in accordance with the methods of Lopez et al. [18]. The meals consisted of 200 g of corn cream potage (Nagoya Seiraku Co., Ltd., Nagoya, Japan), 37 g of salt-free butter (Megmilk Snow Brand Co., Ltd., Tokyo, Japan), and 29 g of meal test C (Saraya Co., Ltd., Osaka, Japan); total amount of fat in the meals was 47.2 g. After a 12-h fasting period, the subjects ingested the OFLT meals and concomitantly consumed the test FM. Blood samples were collected from the median cubital, basilic, or cephalic vein before intake of the OFLT meals (fasting) and each hour after 8 h of OFLT meal intake.

### Blood sample analyses

The blood samples were centrifuged at 1 730 × *g* for 10 min at 4°C, and the supernatant was stored at a temperature lower than −30°C until analysis. Postprandial serum NEFA and TAG levels were measured at 0 (fasting), 60, 120, 180, 240, 300, 360, 420, and 480 min. Other blood parameters were measured at fasting state. Assays of serum NEFA, TAG, total cholesterol, low-density lipoprotein cholesterol, high-density lipoprotein cholesterol, insulin, amylase, total protein, alkaline phosphatase, aspartate aminotransferase, alanine aminotransferase, lactase dehydrogenase, gamma-glutamyl transpeptidase, total bilirubin, plasma glucose, and blood hemoglobin A1c (HbA1c) levels were performed at BML (Bio Medical Laboratories) Inc. (Tokyo, Japan). Serum apolipoprotein B-48 (apo B-48) levels were determined using an ELISA kit (AKHB48J, Shibayagi Co., Ltd., Gunma, Japan), in accordance with the manufacturer’s instructions. Briefly, sera diluted 500- or 1000-fold in the assay diluents were added to microtiter wells coated with immobilized anti-apo B-48 monoclonal antibody and incubated at room temperature for 1 h. After washing with a washing buffer, biotin-conjugated anti-apo B-48 was added to each well and incubated with gentle shaking at room temperature for 1 h. After the plate was washed, horseradish peroxidase-conjugated avidin solution was added, and the resultant solution was incubated at room temperature for 30 min. After the plate was washed, chromogenic substrate solution was added, and the resultant solution was incubated at room temperature for 20 min. Finally, a stop solution for the reaction was added, and then the absorbance of 450 nm at each well was measured using a plate reader.

### Statistical analysis

The baseline characteristics of the subjects were expressed as means with their standard deviations. Other data were expressed as means with their standard errors. The incremental area under the curve (iAUC) for NEFA and TAG were calculated using the trapezoidal rule. Differences in the amount of change between the control and active FM periods were analyzed using a paired *t* test. Postprandial serum TAG levels were determined by a one-sided test because in our previous study, LG2055 inhibited the absorption of dietary TAG in rats, making it unnecessary to consider lipid absorption enhancement [4]. The statistical differences in the other parameters were analyzed by a two-sided test. We considered a *P* < 0.05 to be statistically significant.

## Results

### Effects of the active FM on the postprandial NEFA and TAG levels

**Figure 1:**
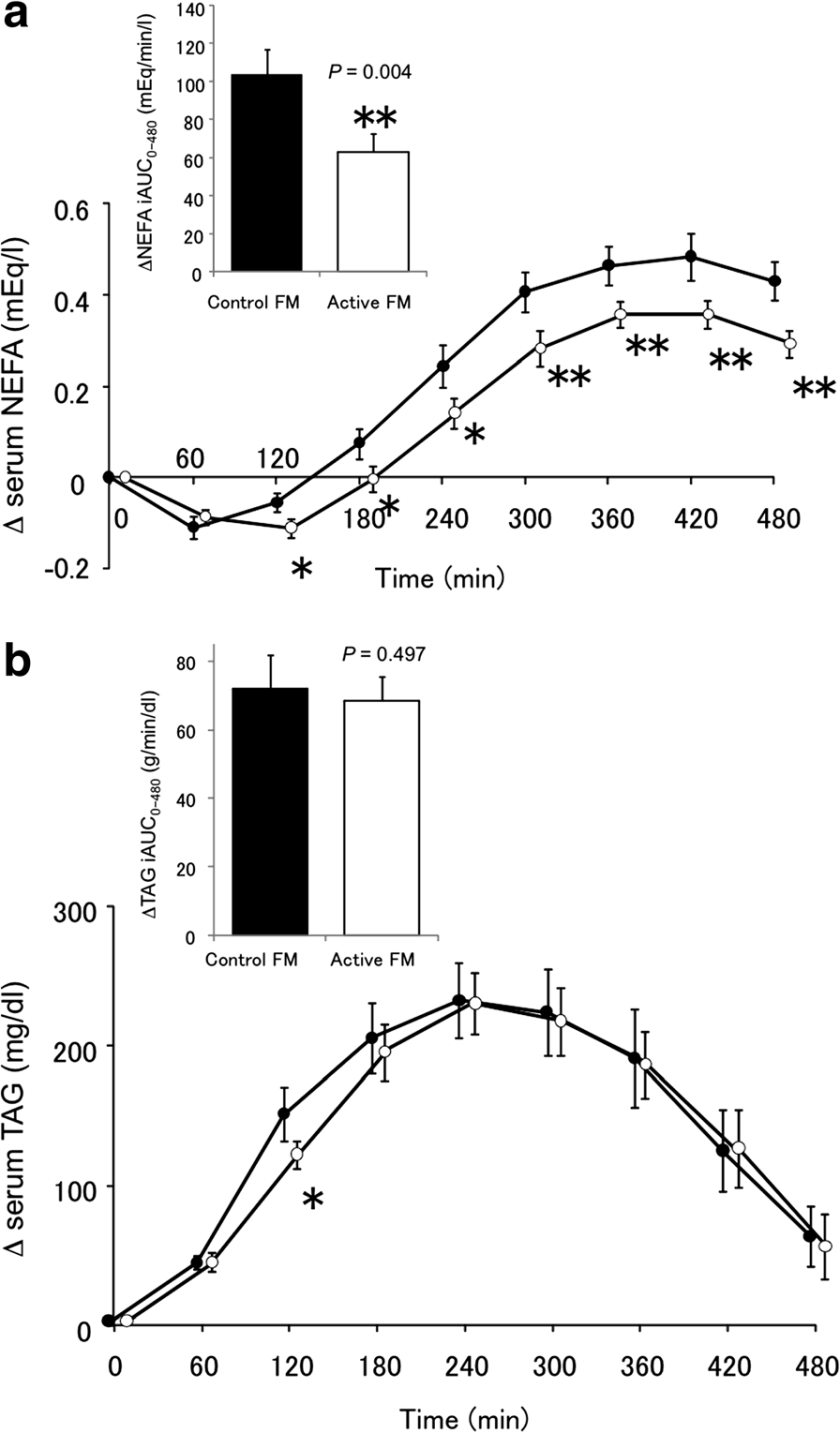
**The changes in the postprandial serum NEFA (a) and TAG levels (b) in response to the oral fat-loading test (OFLT) meal.** The black circles indicate the level after the intake of the control FM, and the white circles indicate the level after the intake of active FM. The incremental areas under the curves (iAUC) are shown as inserts. Values are expressed as mean (SE). *P < 0.05, **P < 0.01, versus the control FM. Abbreviations: NEFA, non-esterified fatty acid; TAG, triacylglycerol; FM, fermented milk.

All the participants followed the study protocol without difficulty. The postprandial responses of serum NEFA level after the intake of the OFLT meals are shown in Figure 1a. The serum NEFA levels showed a temporary downturn from the baseline and then turned upward. Active FM significantly decreased the postprandial serum NEFA levels from 120 to 480 min after the intake of the OFLT meals compared with the control FM (from 120 to 240 min, *P* < 0.05; from 300 to 480 min, *P* < 0.01), leading to a significantly lower NEFA-iAUC (*P* < 0.01). There was no significant difference in NEFA-iAUC between males and females (99.4 ± 16.2 mEq/min/l and 115.6 ± 23.3 mEq/min/l in the control FM period, respectively; 61.4 ± 12.1 mEq/min/l and 75.8 ± 13.2 mEq/min/l in the active FM period, respectively).

The postprandial responses of the serum TAGs are illustrated in Figure 1b. When the subjects consumed the OFLT meal, the serum TAG levels quickly increased from the baseline (0 min) and reached their highest levels at 240 min after the OFLT meal intake. In the active FM period, the serum TAG level at 120 min after the intake of the OFLT meals was significantly lower (*P* < 0.05) than that in the control FM period. No significant difference in TAG-iAUC was found between the periods.

### Effects of the active FM on the anthropometric and fasting blood parameters

The effects of the control and active FM preparations on the anthropometric and fasting blood parameters are listed in Table 2. None of the anthropometric parameters showed statistically significant differences in changes from W0 between the control and active FM periods. Among the blood parameters, the value of change from W0 in fasting serum NEFA level in the active FM period significantly decreased (*P* < 0.001) compared with that in the control FM period. There was no significant difference in the value of change from W0 in fasting NEFA level between males and females (0.09 ± 0.05 mEq/l and 0.10 ± 0.07 mEq/l in the control FM period, respectively; −0.06 ± 0.04 mEq/l and −0.04 ± 0.01 mEq/l in the active FM period, respectively). A significant difference (*P* = 0.002) in the value of change from W0 in HbA1c level was observed between the control and active FM periods, in which the value was greater in the active period than in the control period (Table 2), although these real values were lower than 6.5% of the cutoff HbA1c level for diabetes diagnosis as recommended by the World Health Organization [20]. No significant differences in the other fasting serum parameters were found between the control and active FM periods.

**Table 2:** Anthropometric parameters and fasting levels of the blood parameters before and after the intake of control or active FM

**Parameters**	**Period**	**W0**		**W4**		**Change from W0**		***P *** **value**
**Anthropometric parameters**								
Body weight	Control	70.5	(2.4)	70.3	(2.4)	−0.23	(0.26)	0.093
(kg)	Active	70.7	(2.4)	70.6	(2.3)	−0.04	(0.12)	
BMI	Control	24.5	(0.5)	24.4	(0.5)	−0.09	(0.09)	0.512
(kg/m^2^)	Active	24.5	(0.5)	24.5	(0.5)	−0.01	(0.04)	
Waist	Control	87.4	(1.5)	85.6	(1.3)	−1.78	(0.53)	0.461
(cm)	Active	85.4	(1.5)	84.7	(1.4)	−0.75	(0.35)	
**Blood parameters**								
TAG	Control	246.5	(18.9)	272.4	(26.9)	25.9	(24.9)	0.528
(mg/dl)	Active	268.3	(31.5)	273.4	(33.5)	5.2	(14.4)	
NEFA	Control	0.382	(0.033)	0.472	(0.034)	0.090	(0.037)	0.0003
(mEq/l)	Active	0.399	(0.031)	0.344	(0.026)	−0.056	(0.026)	
Apo B-48	Control	9.38	(1.44)	9.98	(1.31)	0.59	(0.76)	0.173
(μg/ml)	Active	10.24	(2.06)	9.25	(1.46)	−0.98	(0.89)	
Total cholesterol	Control	215.7	(9.1)	220.5	(8.6)	4.8	(4.3)	0.797
(mg/dl)	Active	214.1	(7.0)	217.2	(7.8)	3.1	(4.4)	
LDL cholesterol	Control	128.7	(8.3)	132.5	(7.1)	3.8	(4.0)	0.347
(mg/dl)	Active	127.0	(7.0)	125.0	(7.3)	−2.0	(3.4)	
HDL cholesterol	Control	43.5	(2.0)	43.4	(2.1)	−0.2	(0.8)	0.504
(mg/dl)	Active	43.6	(2.4)	42.4	(2.2)	−1.2	(1.2)	
Glucose	Control	94.5	(2.0)	94.1	(2.0)	−0.4	(1.0)	0.883
(mg/dl)	Active	93.4	(2.6)	93.4	(2.0)	0.0	(1.9)	
Insulin	Control	9.01	(0.66)	8.60	(0.85)	−0.41	(0.46)	0.998
(μU/ml)	Active	10.63	(2.39)	10.22	(0.95)	−0.41	(2.45)	
Amylase	Control	81.5	(8.3)	94.3	(21.1)	12.8	(14.5)	0.217
(U/l)	Active	103.5	(21.4)	91.5	(15.3)	−12.0	(8.6)	
HbA1c	Control	5.04	(0.04)	5.01	(0.05)	−0.03	(0.03)	0.002
(%)	Active	5.05	(0.05)	5.13	(0.05)	0.08	(0.01)	
Total protein	Control	7.05	(0.07)	7.14	(0.06)	0.09	(0.05)	0.192
(g/dl)	Active	6.99	(0.07)	7.00	(0.07)	0.01	(0.04)	
ALP	Control	215.9	(13.0)	219.3	(13.5)	3.4	(4.6)	0.421
(U/l)	Active	209.3	(12.3)	227.5	(20.8)	18.2	(16.2)	
AST	Control	20.8	(1.2)	20.7	(1.2)	−0.1	(0.8)	0.974
(U/l)	Active	21.0	(1.2)	21.0	(1.1)	0.0	(0.9)	
ALT	Control	24.8	(1.8)	24.2	(2.3)	−0.6	(1.7)	0.674
(U/l)	Active	23.2	(2.1)	23.9	(1.9)	0.7	(1.7)	
LDH	Control	159.9	(3.4)	159.3	(3.0)	−0.6	(1.8)	0.055
(U/l)	Active	154.4	(2.9)	161.2	(4.2)	6.8	(2.6)	
γ-GTP	Control	49.2	(7.5)	52.8	(7.0)	3.7	(3.0)	0.484
(U/l)	Active	48.3	(7.1)	61.6	(17.3)	13.4	(12.3)	
Total bilirubin	Control	0.82	(0.09)	0.81	(0.06)	−0.01	(0.06)	0.088
(mg/dl)	Active	0.88	(0.06)	0.71	(0.06)	−0.18	(0.04)	

*Abbreviations*: *TAG* triacylglycerol, *NEFA* non-esterified fatty acid, *Apo* apolipoprotein, *LDL* low-density lipoprotein, *HDL* high-density lipoprotein, *HbA1c* hemoglobin A1c, *ALP* alkaline phosphatase, *AST* aspartate aminotransferase, *ALT* alanine aminotransferase, *LDH* lactase dehydrogenase, *γ-GTP* gamma-glutamyl transpeptidase. The data are expressed as mean (SE) at each time point before (week 0, W0) and after (week 4, W4) intake of control or active FM (n = 20). A paired *t* test was performed to compare the changes in the response to the control and active FM.

### Daily life and adverse events

Irregularities in daily life or adverse events related to FM consumption were not observed throughout the course of the study, according to the daily record and interview with the physician (data not shown).

## Discussion

We investigated the postprandial lipid responses after an OFLT meal intake in the subjects with hypertriacylglycerolemia who ingested fermented milk with or without LG2055 for 4 weeks. To our knowledge, this is the first intervention study on OFLT in probiotics. The postprandial serum NEFA levels from 120 to 480 min after OFLT meal intake in the active FM period were significantly lower than those in the control FM period (Figure 1a). The postprandial serum TAG levels in the active FM period tended to be lower from 60 to 180 min and significantly lower at 120 min compared with the control FM period (Figure 1b). This time point of 120 min corresponded to the time point when the postprandial NEFA levels in the active FM period were still prevented from increasing, whereas in the control FM period, the NEFA levels already began to increase from the time point of 60 min. We consider that this delayed onset of the increase in the NEFA level in the active FM period might lead to the subsequent lowered NEFA levels in the active FM period compared with the control FM period. Furthermore, the plateau level of the postprandial NEFA that was attained in the active FM period at approximately 360 min was significantly lower than that in the control FM period at around the same time point; thus, we also consider that the lower NEFA levels reflected the inhibited increase in the TAG levels, which are primarily influenced by the state of fat absorption.

Tan et al. [12] also reported that postprandial NEFA levels from 120 to 240 min and postprandial TAG level at 120 min after OFLT meal intake in diabetes subjects treated with orlistat were significantly decreased compared with those in subjects treated with a placebo. They described that the decreased NEFA levels were generated from the event that NEFA was not supplied from the postprandial TAG owing to the suppression of fat absorption. This finding is consistent with our results and suggests that the suppression of lipid absorption by LG2055 may contribute to the significantly decreased NEFA levels in this postprandial period. Inhibition of pancreatic lipase can possibly suppress lipid absorption, as it has recently been reported that two *Lactobacilli* strains, viable *Lactobacillus gasseri* NLB367 [21] and heat-killed *Lactobacillus pentosus* S-PT84 [22], inhibited pancreatic lipase in vitro, although whether our LG2055 strain inhibits lipase activity has yet to be examined.

The postprandial serum NEFA levels after 240 min (the late postprandial phase) in the active FM period were significantly lower than those in the control FM period, although the postprandial TAG levels during the time did not differ between the control and active FM periods. NEFA is known to be produced from the TAG hydrolysis process by lipoprotein lipase to produce NEFA and glycerol, which are incorporated into the liver, muscles, and adipose tissues. In the present study, TAG was likely to be equally hydrolyzed to generate the same amount of NEFA, given that TAG levels did not differ between the control and active FM periods after 240 min. However, difference in the ability to incorporate NEFA could cause the different NEFA levels between the control and active FM periods; thus we consider that the control FM period might less incorporate NEFA than the active FM period. Such NEFA which is not incorporated and remaining in the bloodstream is known to be crucial for the late postprandial rise in NEFA [23,24].

Ingestion of the anti-diabetic drug rosiglitazone, known as a peroxisome proliferator-activated receptor (PPAR)-γ agonist, was reported to significantly lower plasma NEFA levels during the postprandial period and simultaneously reduce plasma TAG levels from 240 to 360 min [25]. However, LG2055 did not affect serum TAG levels from 240 to 480 min (Figure 1b). Rosiglitazone ameliorates blood glucose levels in diabetic subjects via the incorporation of some amount of blood glucose into the adipose tissues, along with some amount of blood NEFA derived from the TAG hydrolysate; this mechanism occasionally leads to adverse effects such as increases in body fat mass and body weight [26,27]. By contrast, while LG2055 ingestion decreased body fat mass and body weight in humans [8,9], postprandial NEFA levels were also significantly lowered by LG2055 ingestion. Thus, the effects of LG2055 on lipid metabolism may differ from those of the PPAR-γ agonist.

Judging from the unchanged TAG-iAUC result, we consider the magnitude of the fat absorption suppression by LG2055 to be mild (Figure 1b). The mild suppression may be due to the experimental condition we applied in the present study. Although there are no official guidelines for the OFLT, recent studies performed OFLT using a moderate (0.35-0.7 g of fat/kg body mass) rather than a high-fat meal [28], and our study has also been carried out within the moderate range (0.67 g of/kg body mass). In addition to the amount of fat loading, other factors such as racial differences greatly affect the postprandial TAG response [29]. Therefore, we compared the experimental condition of our study with that of other studies, in which Japanese subjects were recruited and the effects of functional food components on the postprandial TAG levels were examined [30-32]. The amount of fat loading was higher, and the high baseline fasting serum TAG levels of our subjects, in comparison with those of other studies. Other studies performed an OFLT and evaluated the suppressive effects of functional food components [30-32] at loading levels of 18.8 to 40 g of fat, whereas our study was performed at a loading level of 47.2 g of fat. In addition, the baseline fasting serum TAG levels of the subjects in the other studies ranged from 144.5 to 175 mg/dl, whereas the mean baseline level in our study was 277.0 mg/dl. These factors might lead to higher increases in TAG after OFLT; the ΔTAG level was 231.2 mg/dl in our study, whereas it ranged from 61 to 130 mg/dl in the other reports. Thus, the great increase in postprandial TAG levels in our study might have obscured the suppressive effect of LG2055 on postprandial TAG responses.

An elevation of blood TAG levels is a well recognized cardiovascular risk factor [33]. We consider that lowering NEFA levels is also beneficial for human health as well as lowering TAG levels. Elevated NEFA levels are a predictor of subsequent development of type 2 diabetes [34], and associated with the deterioration of glucose tolerance [35,36]. Dysregulation of plasma NEFA levels is central to the insulin resistance and is associated with dyslipidemia [37]. In the present study, a significant decrease in fasting NEFA level at W4 from W0 was observed in the active FM period (Table 2), which could be a possible accumulated effect of the decrease in the postprandial NEFA levels (Figure 1a) over time. The ability of LG2055 to continuously lower serum NEFA levels may help reduce the risk for T2DM and obesity.

Though physiologically normal, a significant difference in the value of change from W0 in HbA1c level was observed between the active and control FM periods, in which the value was greater in the active FM period than in the control FM period (Table 2). Additionally, within-period comparisons between W0 and W4 did not reveal significant differences both in the control and in the active FM periods (statistical symbols were not shown). This observation is consistent with that reported by Chang et al. [38]. They reported that blood HbA1c levels tended to increase in healthy volunteers who consumed a probiotic yogurt NY-YP901 for 8 weeks, whereas the intake of placebo yogurt for 8 weeks tended to decrease blood HbA1c levels.

## Conclusions

In conclusion, the consumption of probiotic LG2055 reduced postprandial serum NEFA levels after the intake of OFLT meals and fasting NEFA levels at the end of the feeding period, suggesting its possible contribution to the reduction of the risk for obesity and T2DM.

## Abbreviations

LG2055: Lactobacillus gasseri SBT2055; OFLT: Oral fat-loading test; FM: Fermented milk; NEFA: Non-esterified fatty acid; TAG: Triacylglycerol; BMI: Body mass index; MOS: Mannooligosaccharide; HbA1c: hemoglobin A1c; apo B-48: Apolipoprotein B-48; iAUC: Incremental area under the curve.

## Competing interests

The authors declare that they have no competing interest.

## Authors’ contributions

AO and BS wrote the manuscript. YK assisted with the data analysis and interpretation. KK was responsible for the study. MS supervised the study design and commented on the paper. All authors read and approved the final manuscript.
